# Acute Rheumatism—Pericarditis—Hypertrophia Cordis, &c.

**Published:** 1831-01-01

**Authors:** 


### REGIMENTAL HOSPITAL, LIFE GUARDS.

ACUTE RHEUMATISM—PERICARDITIS—HYPERTROPHIA CORDIS, &c.

*Case.* A young recruit became affected with acute rheumatism of severe type in the year 1826, and was received into hospital with the usual symptoms, on the 3d of April. On the 7th of the same month, there was an evident extension of the disease to the chest, as evinced by pain under the sternum, cough, and full strong pulse, &c. These symptoms were relieved by depletion—and on the 15th we find him "gaining ground daily, pulse 76." On the 19th, however, the pulse was 130—the swelling of the wrist subsided—pain at the sternum, with cough upon inspiration. Depletion—and next day the symptoms were moderated. 25th. Pulse 130—pain in the left side, without cough. Leeches, colchicum, &c. On the 27th, intermission of the pulse was first noticed, with pain periodically recurring in the region of the heart. This organ was found to beat strongly under the hand—some incoherency of ideas obtained. After some variations, we find him on the 24th June, convalescent, with a pulse at 60—but still, "the hand applied to the region of the heart produced a sensation as if the cardiac motions were impeded by adhesions of the pericardium, and the heart itself was greatly enlarged." As our good friend Dr. Gregory was assisting Mr. Broughton with his valuable advice at this time, we congratulate him on having such a highly sensible stethoscope in the palm of his hand, by which he was enabled to vie with, if not excel any auscultator in the world, not even excepting Laennec!

The man was now put to easy duty, and we are assured by Dr. Gregory that "he continued in good health nearly four years." If a man with a fatal and growing hypertrophy of the heart can be pronounced in good health, the foregoing statement is correct; but we apprehend that "good health" is not the necessary accompaniment of non-complaint. Be that as it may, the man was found, on the 6th of May, 1830, in profound apoplexy, from which he was rescued by very vigorous depletion. Hemiplegia was the sequence of this attack, and, on the 28th July, he was removed, with his regiment, to Brighton, by which removal he was rendered much worse. His heart beat strongly—his tongue was loaded and brown—and the pulse was much accelerated. From these symptoms, however, he partially emerged; but still experienced "severe fits of dyspnœa and rapid action of the heart." He sunk into a state of imbecility, and died on the 27th of August last.

*Dissection.* The usual apoplectic cyst was found in one of the hemispheres of the brain, containing two ounces of a thick curdy fluid. On opening the thorax, the heart appeared to occupy a considerable portion of its cavity, with universally adherent pericardium. The right chambers were sound—the parietes of the left chambers were much thickened. The pleura costalis and pulmonalis adhered by elongated bands of coagulable lymph. The liver was granulated. The heart weighed one pound and three quarters.*

*Remarks.* The above case is chiefly interesting as shewing the length of time which an ultimately fatal hypertrophy of the heart will run before death is produced. It also illustrates that now recognized principle in pathology, that hypertrophy of the heart has a strong tendency to induce apoplexy. It is not at all likely that so young a man would have had the latter disease, if the former had not existed for a considerable time. The paper is headed "Pathology of Rheumatism;" but we think this title is somewhat questionable. Hypertrophy of the heart is no infrequent sequence of acute rheumatism—but does not necessarily acknowledge that disease for its cause or origin. We conceive then that a case of hypertrophia cordis is not a discourse on the nature of rheumatism, which pathology of rheumatism must mean. It would not appear very congruous to head a case of enlarged spleen—"Pathology of Fever—or phlegmatia dolens, as "PATHOLOGY OF PREGNANCY." Rheumatism is more connected with the etiology than the pathology of cardiac inflammation and hypertrophy. These, however, are only trifling verbal criticisms. The case itself is well worthy of consideration.

* Analyzed from the Med. Gazette.

